# Photothermolysis with 1550 nm Fractional Laser Promotes Regeneration of Gingival Mucosa

**DOI:** 10.3390/bioengineering12111180

**Published:** 2025-10-29

**Authors:** Elena Morozova, Alexey Fayzullin, Polad Osmanov, Anna Timakova, Peter Timashev, Svetlana Tarasenko

**Affiliations:** 1 Department of Propaedeutics of Dental Diseases, Peoples’ Friendship University of Russia Named After Patrice Lumumba, 6 Miklukho-Maklaya St., 117198 Moscow, Russia; morozova_elan@pfur.ru; 2 Institute for Regenerative Medicine, I.M. Sechenov First Moscow State Medical University (Sechenov University), 8/2 Trubetskaya St., 119991 Moscow, Russia; timakova_a_a@staff.sechenov.ru (A.T.); timashev_p_s@staff.sechenov.ru (P.T.); 3 E.V. Borovsky Institute of Dentistry, I.M. Sechenov First Moscow State Medical University (Sechenov University), 8/2 Trubetskaya St., 119991 Moscow, Russia; poladosmanov8@gmail.com (P.O.); tarasenko_s_v@staff.sechenov.ru (S.T.)

**Keywords:** dentistry, oral mucosa, periodontal tissues, regeneration, laser, fractional laser photothermolysis

## Abstract

Fractional laser photothermolysis, long established in dermatology, enables controlled microthermal injury that stimulates repair without scarring, but its potential in oral tissue regeneration has not been systematically explored. In this study, we conducted the first controlled experimental evaluation of a 1550 nm erbium fiber laser for oral mucosa regeneration. Thirty-two rabbits underwent fractional photothermolysis at energy levels of 70, 100 and 130 kJ, with gingival biopsies collected at 1, 14, 28 and 42 days for histological and immunohistochemical assessment of epithelial repair, stromal remodeling, inflammation and angiogenesis. All energy modes produced microcoagulation columns followed by progressive epithelial thickening, fibroblast proliferation and neoangiogenesis. The 70 kJ mode occasionally led to residual fibrosis, whereas higher energies (100–130 kJ) promoted effective connective tissue remodeling and de novo tissue formation without scarring. Complete epithelial recovery occurred within two weeks, indicating a safe and optimal interval for repeated exposure. Overall, the results demonstrate that 1550 nm fractional photothermolysis is a safe and effective method to induce regenerative responses in oral tissues, establishing a foundation for its translational application in periodontal and peri-implant regeneration.

## 1. Introduction

Despite being extensively studied, inflammatory periodontal diseases remain a major challenge in dentistry. Epidemiological data indicate that the prevalence of chronic inflammatory diseases of the periodontal tissues among adults ranges from 20% to 98% [1,2,3,4]. The number of affected patients is further increasing due to the growing incidence of peri-implantitis [5,6,7]. According to the 2016 Global Burden of Disease Study, severe periodontal disease ranked 11th in prevalence among all medical conditions worldwide [2]. Importantly, periodontal diseases are increasingly diagnosed in younger populations and are frequently observed in patients under 40 years of age [8]. Their global prevalence is also expected to rise due to demographic shifts, including population aging and reduced tooth loss in the elderly [9,10].

The role of keratinized gingiva in maintaining periodontal health was first highlighted by Lang and Löe in 1972. Their findings demonstrated that keratinized gingiva less than 2 mm in width predisposes to inflammatory disease, even in the absence of plaque [11]. Subsequent research has shown that lack of keratinized gingiva around dental implants contributes to excessive soft tissue mobility, impaired hygiene and the development of peri-implant pockets that may ultimately result in implant loss [12,13].

Among promising strategies to address this issue is the use of laser-based microcoagulation to increase keratinized gingival volume [14]. Fractional laser photothermolysis, originally established in dermatology, has gained wide clinical application in recent years [15,16]. The therapeutic potential of fractional laser photothermolysis arises from its ability to induce controlled microthermal damage while preserving surrounding viable tissue, thereby stimulating biological tissue regeneration. Compared with other laser methods, non-ablative fractional laser photothermolysis provides precise control over the width, depth and density of microdamage, allowing patient-specific treatment while minimizing adverse effects [17]. A substantial body of dermatological research has confirmed that this treatment supports scar-free healing and effective tissue remodeling, enabling its use in the treatment of atrophic and other scar types [18,19,20,21].

Given its proven efficacy in dermatology and the high regenerative capacity of the oral mucosa, extending fractional laser photothermolysis to dentistry is of considerable interest. Nonetheless, current studies investigating fractional laser photothermolysis in the treatment of inflammatory periodontal diseases remain scarce. Experimental and clinical findings suggest its potential: in a rabbit model, fiber-optic laser coagulation with a 980 nm diode system induced enhanced collagen synthesis and complete mucosal recovery by day 90 without fibrosis [22]. More recently, fractional laser therapy with a 940 nm diode laser was reported to accelerate healing and reduce inflammation in desquamative gingivitis associated with oral lichen planus [23].

With the advent of new lasers, such as those operating at 1550 nm, the investigation of fractional laser photothermolysis for dental applications has gained momentum. The oral mucosa’s superior regenerative capacity compared to skin suggests that fractional laser treatment may effectively stimulate keratinized gingiva formation [24]. However, treatment outcomes depend on carefully optimized parameters to ensure both safety and efficacy, including the size, spacing and arrangement of microcoagulation defects, as well as laser operating mode. The present study was therefore designed to evaluate the response of the oral mucosa to fractional laser photothermolysis under different laser settings in a rabbit model.

## 2. Materials and Methods

### 2.1. Study Design

The experimental study was carried out at the Department of Surgical Dentistry of the E.V. Borovsky Institute of Dentistry and at the Science & Technology Park of Biomedicine, I.M. Sechenov First Moscow State Medical University, Ministry of Health of the Russian Federation (Sechenov University). The study protocol was approved by the Ethics Committee of Sechenov University (Protocol No. 01-22, 20 January 2022).

**Figure 1:**
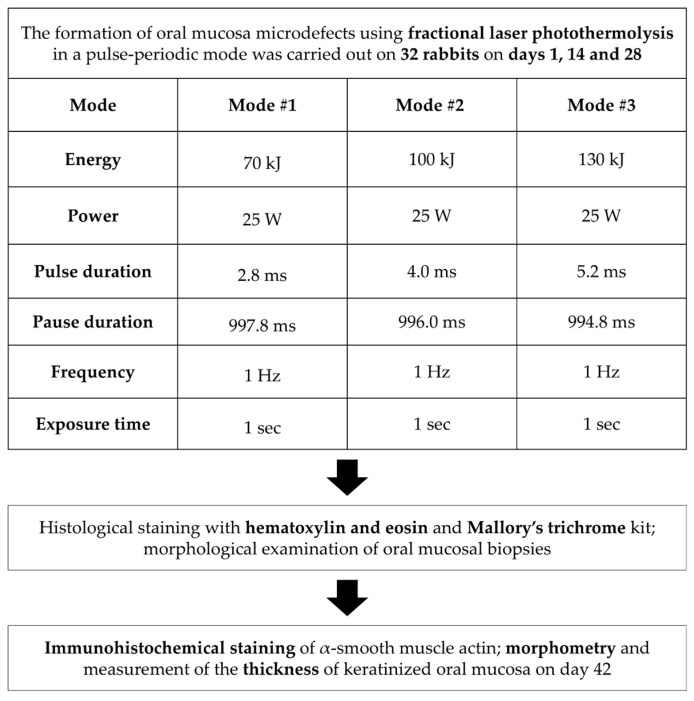
Study design.

A total of 32 adult male grey chinchilla rabbits (3.5–4.0 kg) were used to evaluate the effects of fractional laser irradiation on keratinized oral mucosa in different operating modes of a 1550 nm laser (maximum power 25 W) (Figure 1). Animals were housed in a certified vivarium under standard laboratory conditions in accordance with good laboratory practice for preclinical studies (GOST R50258-92, GOST 351000.3-96, GOST 51000.4-96). The environment was maintained at room temperature with a 12 h light/dark cycle, and animals had free access to pelleted feed and water. Before each procedure, rabbits were weighed and anesthetized by intramuscular administration of Rometar (3 mg/kg) and Zoletil (5 mg/kg).

All experiments were conducted on healthy animals without induced inflammation, as surgical procedures for gingival augmentation are clinically indicated only after complete resolution of local inflammatory processes. This design ensured alignment with real protocols, as the experimental conditions accurately reflected the physiological state (gingival recession without evident inflammation) in which laser- or biomaterial-based soft tissue regeneration is performed in patients.

### 2.2. The Laser Device

Fractional laser treatment was performed using a single-wavelength surgical laser device, LSP–IRE-Polus (No. RZN 2013/850), operating at 1550 nm with a peak power of 25 W. The device was based on a fiber laser module capable of generating continuous, pulsed or pulse-periodic radiation (Table 1). The pulse duration ranged from 2.8 to 8.0 ms (70–200 mJ per pulse). Radiation was delivered to the tissue via a focusing tip equipped with interchangeable lenses, providing beam diameters of 25 or 50 µm (at the e-2 level), with focal lengths of 19 mm and 25.4 mm. The device is designed for non-ablative fractional photothermolysis of soft oral tissues and was operated in facilities equipped for laser surgery.

### 2.3. Surgical Technique

**Figure 2:**
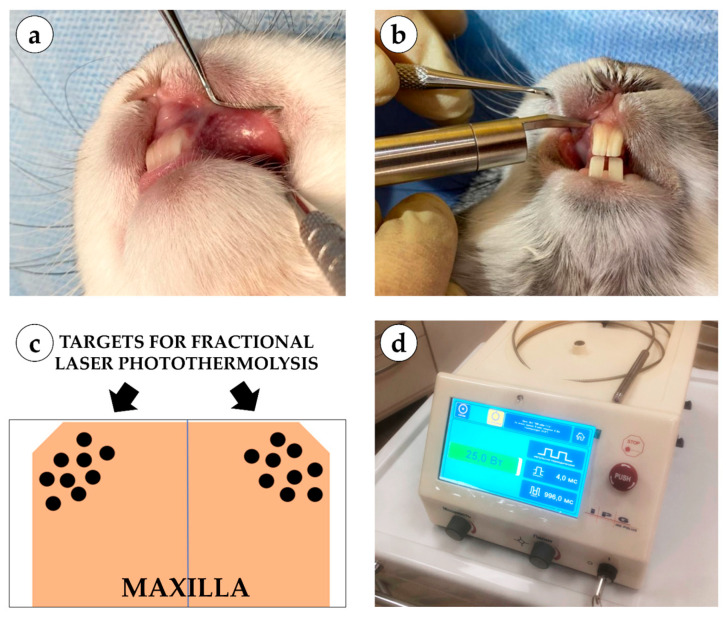
Experimental setup and laser application: (**a**) application of fractional laser during surgery; (**b**) microcoagulation columns on the oral mucosa of a rabbit; (**c**) schematic representation of coagulation column application; (**d**) single-wavelength surgical laser device LSP “IRE-Polus” (1550 nm).

Fractional photothermolysis was applied to the intact mucosa of the alveolar process of the maxilla. On each rabbit, coagulation microdefects were created on opposite sides of the jaw in different laser modes (Figure 2). The laser handpiece was positioned perpendicular (90°) to the mucosal surface, and treatment zones were selected according to the animals’ anthropometric parameters. Microdefects were applied in a uniform distribution, spaced 1.0–1.5 mm apart. Laser irradiation was performed three times per animal at two-week intervals (days 1, 14, and 28).

All oral mucosa biopsies were divided into three study groups depending on the laser mode: Group 1 included biopsies after laser exposure in mode 1: energy 70 kJ, power 25 W, pulse duration 2.8 ms, pause duration 997.8 ms, frequency 1 Hz, exposure time 1 s. In Group 2, the tissues were examined after laser exposure in Mode 2: 100 kJ energy, 25 W power, 4.0 ms pulse duration, 996 ms pause duration, 1 Hz frequency, and 1 s exposure time. The mode in Group 3 used the following parameters: 130 kJ energy, 25 W power, 5.2 ms pulse duration, 994.8 ms pause duration, 1 Hz frequency, and 1 s exposure time.

At each time point (days 1, 14, 28 and 42), eight rabbits were operated on, with both halves of the maxilla exposed to the laser. This design provided 15 experimental samples (five per laser mode) and one untreated control per time point. The distribution of rabbit oral mucosa samples by group and time point is presented in Table 2.

Animals were euthanized following euthanasia guidelines by administering an overdose of Zoletil on days 1 (10 min after laser exposure), 14, 28 and 42. Mucosal biopsies were fixed in 10% neutral buffered formalin for the histological analysis.

### 2.4. Morphological Study and Morphometry

Four-μm-thick sections of the formalin-fixed-paraffin-embedded gingival tissue samples were stained with hematoxylin and eosin (H&E) and Mallory trichrome for the detection of collagen fibers. A LEICA DM4000 B LED microscope, equipped with a LEICA DFC7000 T digital camera running under the LAS V4.8 software (Leica Microsystems, Wetzlar, Germany), was used for the examination and imaging of the samples.

Four-μm-thick sections of the formalin-fixed-paraffin-embedded tissue samples were deparaffinized, incubated with 3% H_2_O_2_ for 10 min, underwent heat induced epitope retrieval (pH 6.0 sodium citrate buffer, 30 min in 80 °C water bath), additionally blocked with Background Block (Cell Marque, Rocklin, CA, USA) and incubated with mouse monoclonal primary antibodies against α-smooth muscle actin (α-SMA) (A2547, Merck, Rahway, NJ, USA, diluted 1:400) and detected by horseradish peroxidase-conjugated secondary goat antibodies (G-21040, Invitrogen, Carlsbad, CA, USA, diluted 1:1000) and diaminobenzidine with hematoxylin counterstaining.

Features of inflammation (exudation, inflammatory infiltration) and regeneration (neoangiogenesis) were evaluated on a 4-point scale (Table 3, Table 4 and Table 5). α-SMA expression at laser treatment sites was assessed using a semi-quantitative system (Table 6). The score systems were taken from published studies on guided tissue regeneration [25,26].

Thickness of mucosa was measured from the basal membrane of epithelium to the muscular or adipose tissue at the center of the treatment area.

The statistical analysis of the experimental data was performed with a standard program package, GraphPad Prism version 10.00 for Windows (GraphPad Software, Inc., San Diego, CA, USA). The intergroup differences in the quantitative data (thickness of mucosa) were analyzed using one-way ANOVA followed by Tukey’s multiple comparison test. The search for the differences in the histological scores was conducted using the Kruskal–Wallis test followed by Dunn’s multiple comparison test. The statistical analysis results were presented as column graphs of the mean values and standard deviations (SD) for quantitative data, and as median values and interquartile range for histological scores. *p*-values equal to or less than 0.05 were considered statistically significant.

## 3. Results

In the control group, gingival tissue was covered with stratified squamous epithelium with distinct papillae. One sample demonstrated focal epithelial atrophy, characterized by thinning, smoothing of papillae and loss of the granular layer. The mucosa consisted of collagen fibers, occasionally hyalinized, with sparse lymphocytes and fibroblasts. Thin-walled capillaries, adipose tissue, nerves, skeletal muscle fibers and mucous glands were also observed. α-SMA expression was weak in vascular smooth muscle (+).

**Figure 3:**
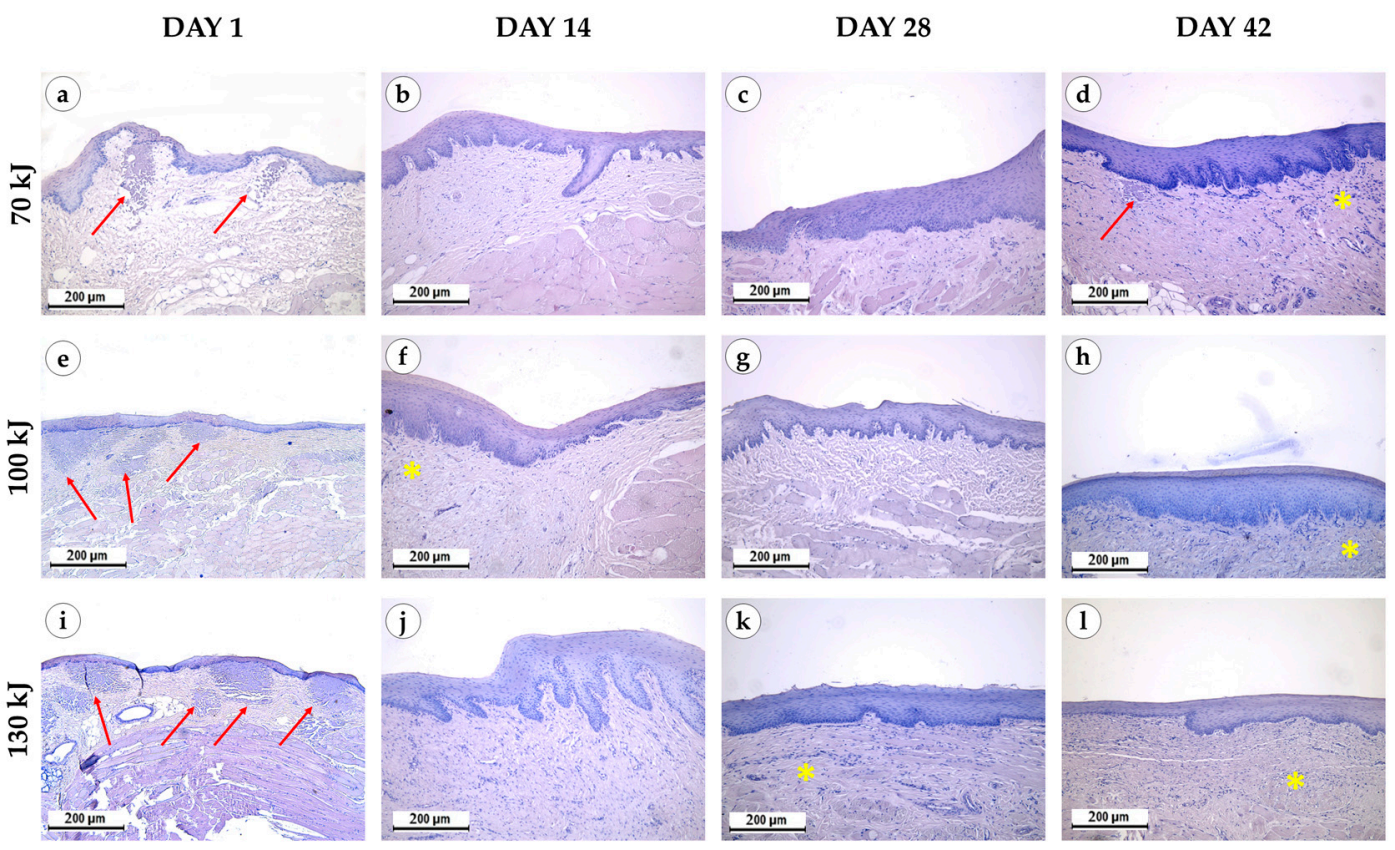
Morphological study of the gingival samples after photothermolysis with 1550 nm fractional laser. The experimental groups were divided by the laser energy 70 kJ (**a**–**d**), 100 kJ (**e**–**h**), 130 kJ (**i**–**l**), stained with hematoxylin and eosin, magnification ×200, arrow—fibrinoid necrosis, star—neoangiogenesis.

**Figure 4:**
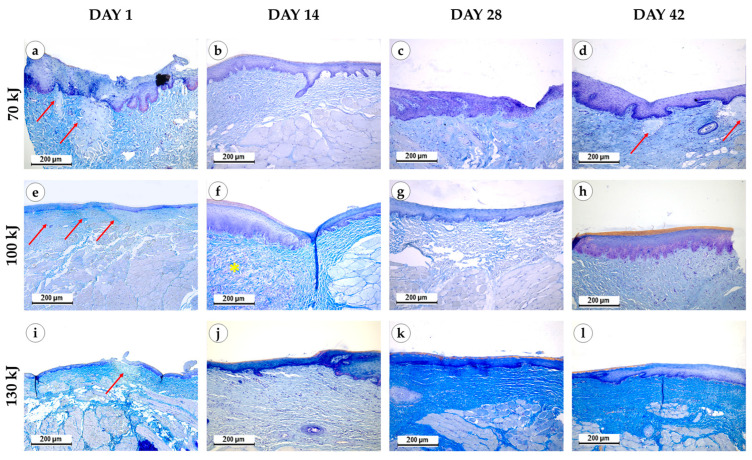
Connective tissue remodeling in the gingival samples after photothermolysis with 1550 nm fractional laser. The experimental groups were divided by the laser energy 70 kJ (**a**–**d**), 100 kJ (**e**–**h**), 130 kJ (**i**–**l**), stained with Mallory’s trichrome kit, magnification ×200, arrow—fibrinoid necrosis, star—myxomatosis.

Immediately after laser treatment, all groups retained epithelial lining with well-defined papillae (Figure 3). In the 70 kJ group, two samples showed stromal myxomatosis, while three exhibited small foci of fibrinoid necrosis surrounded by isolated neutrophils. Mucosal edema and variable collagen disintegration were present, with fibers ranging from bright to pale blue on Mallory staining (Figure 4). In the 100 kJ group, four of five samples demonstrated multiple irregular foci of fibrinoid necrosis and more pronounced edema, though without myxomatosis. The 130 kJ group showed necrosis in two of five samples, with several small, irregular foci. Across all laser settings, mucosal edema, fiber disintegration and mild lymphocytic infiltration were noted, while neutrophil infiltration remained limited.

Two weeks after treatment, epithelial thickening with basal cell proliferation was evident in all groups compared with controls and baseline. Residual fibrinoid necrosis persisted in one sample of the 70 kJ group, but not in the 100 or 130 kJ groups. The 100 kJ group exhibited myxomatosis in two of five samples and evidence of neoangiogenesis in three, whereas the 70 kJ group lacked these features. In the 130 kJ group, fibroblast proliferation was more pronounced than in the control and 70 kJ groups. Mucosal lymphocytic infiltration was mild across all groups, with no evidence of acute inflammation.

Four weeks after treatment, epithelium remained thickened in all groups. In the 70 kJ group, necrosis was absent, but neoangiogenesis was observed in two samples. In the 100 kJ group, all samples showed uniform epithelial thickening, with neoangiogenesis in three and occasional fibroblast proliferation. In the 130 kJ group, lymphocytic infiltration persisted, with neoangiogenesis in four of five samples and fibroblast proliferation in one. No edema or myxomatosis was seen at this stage.

**Figure 5:**
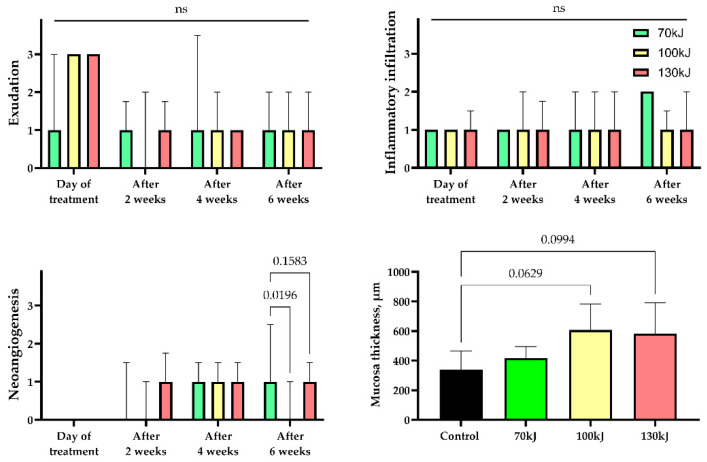
Statistical analysis of histological features of inflammation and regeneration in areas of laser treatment. Exudation, inflammatory infiltration, neoangiogenesis—median values ± interquartile range. Thickness of mucosa—mean ± SD, n.s.—not significant.

**Figure 6:**
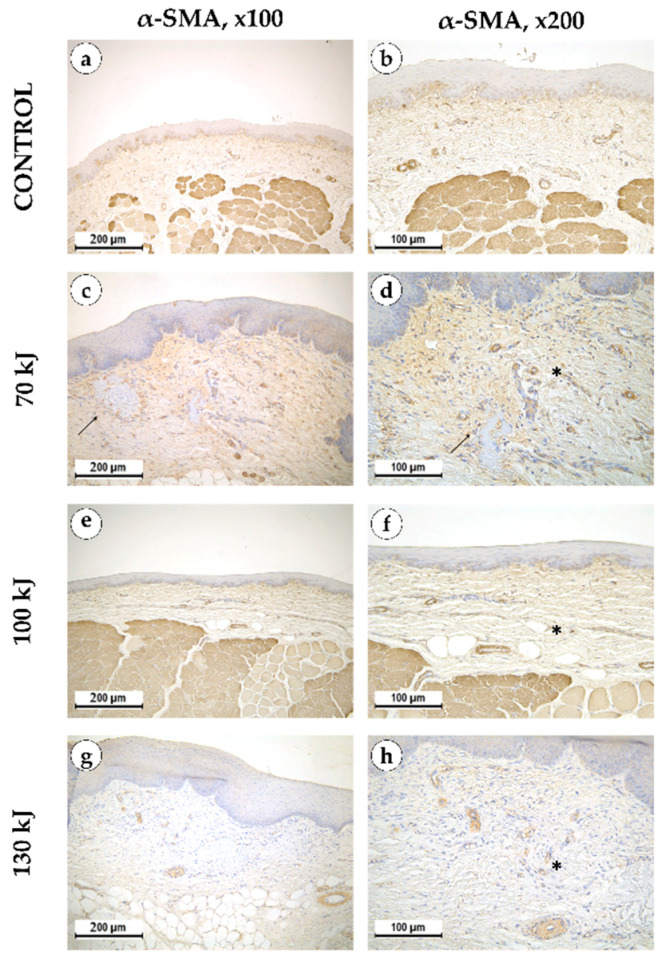
α-smooth muscle actin in intact gingical tissue (**a**,**b**) areas treated with 70 kJ (**c**,**d**), 100 kJ (**e**,**f**) and 130 kJ (**g**,**h**) lasers six weeks after treatment. Immunohistochemical reaction with antibodies against α-smooth muscle actin with hematoxylin counterstaining, magnification ×100 (**left column**), ×200 (**right column**), arrow—healing laser treatment area, star—neoangiogenesis.

Six weeks after treatment, epithelial thickening was consistently observed in all groups, exceeding that of the control specimens (Figure 5). In the 70 kJ group, small foci of fibrinoid necrosis were surrounded by dense fibroblastic proliferation and fibrotic tissue, accompanied by marked neoangiogenesis (*p* = 0.0196 compared with control and 100 kJ). In the 100 kJ group, neoangiogenesis was identified in two of five samples, with a statistical trend toward mucosal thickening (*p* = 0.0629). In the 130 kJ group, connective tissue remodeling was more advanced, with thick collagen bundles, active fibroblast proliferation and de novo connective tissue formation without scarring. Neoangiogenesis was present in three of five samples, and there was a trend toward mucosal thickening (*p* = 0.0994). Across groups, α-SMA expression was moderate in fibroblasts and vascular smooth muscle (++), reflecting maturation of granulation tissue (Figure 6).

## 4. Discussion

To the best of our knowledge, this is the first controlled experimental study to investigate the effects of a 1550 nm fractional Er fiber laser on oral mucosa in dentistry. Previous work in the field evaluated ex vivo porcine tissues and a small clinical series of two patients, demonstrating that laser parameters such as pulse energy, beam diameter and beam divergence could reliably produce microcolumns up to 800 μm in depth and suggesting the feasibility of fractional systems for gingival regeneration [27]. Our study expands on these preliminary findings by employing a controlled in vivo animal model, systematically comparing different energy settings and assessing histological outcomes over multiple time points. This approach provides the first comprehensive evidence for the regenerative potential and safety profile of 1550 nm fractional laser photothermolysis in oral tissues and establishes the foundation for continuing this line of research.

Studies on non-ablative fractional laser treatment of human skin describe a variety of approaches using lasers of different wavelengths and operating parameters. Central to these approaches is the creation of microthermal zones with controlled width, depth and density of microdamage, enabling treatment to be tailored to individual patient needs [15,16,17,20]. By optimizing the ratio between damaged and surrounding healthy tissue, fractional laser photothermolysis in dermatology has been shown to induce regeneration without scar formation, supporting complete tissue recovery [18,19,20,21]. Histological studies have further demonstrated that fractional laser therapy initiates distinct healing phases but may also lead to adverse effects such as intradermal fibrosis and hypopigmentation if parameters are not properly controlled [17,28,29].

In the oral cavity, a minimally invasive microsurgical approach to laser microcoagulation allows for full restoration of the mucosal surface without scarring [22]. Moreover, oral mucosa demonstrates faster inflammatory and epithelialization phases compared with skin wounds, suggesting an inherently superior regenerative capacity [30]. Building on these insights, the present study aimed to assess oral mucosal healing after single and repeated sessions of fractional laser photothermolysis at different energy settings and time intervals, with particular attention to potential inflammation-related side effects.

In the present study, on the day of treatment, all energy modes induced fibrinoid necrosis and edema. At 70 kJ, necrotic foci appeared as large, irregular lesions, whereas 100 and 130 kJ exposures produced multiple smaller foci. Inflammatory responses varied: in the 70 kJ group, inflammation was evident around necrotic regions and occasionally progressed to fibrosis, whereas by six weeks in the 130 kJ group, inflammation had largely subsided without scarring. The persistence of fibrosis only in the 70 kJ group may reflect differences in tissue damage patterns or longer persistence of necrotic tissue.

Proliferative changes, including fibroblast activation, epithelial thickening and neoangiogenesis, were more pronounced at 100 and 130 kJ than at 70 kJ. However, while the 100 kJ setting elicited more pronounced inflammation and carried a greater risk of fibrotic scarring, the 130 kJ setting was associated with reduced inflammatory activity and enhanced regenerative remodeling, with evidence of de novo tissue formation. These results suggest that higher energy levels, when applied fractionally, may better support regenerative outcomes while minimizing fibrotic side effects. Nevertheless, long-term studies are needed to evaluate postoperative volume stability and potential tissue loss.

An additional key observation is the time course of healing. A single session of fractional photothermolysis resulted in complete epithelial recovery and stromal remodeling within two weeks. This finding implies that repeated sessions should be scheduled no earlier than two weeks after the initial procedure, allowing sufficient healing time and reducing the risk of complications related to incomplete epithelial or stromal regeneration.

## 5. Conclusions

The photothermolysis with 1550 nm fractional laser promotes regeneration of the oral mucosa by reducing inflammatory responses, restoring microcirculation and accelerating wound healing. The procedure improves gingival tissue biotype while remaining minimally invasive, painless and cost-effective, requiring no additional pharmacological support. Taken together, these features highlight its potential as a promising therapeutic modality for inflammatory and degenerative periodontal diseases.

## Figures and Tables

**Table 1:** Technical characteristics of the LSP laser-“IRE-Polus” (1.55 µm/25 W).

Parameter	Value
Wavelength of the first working radiation, λ1 μm	1.55
Value according to specifications, W, not less than	2.0
Actual value for λ1, W	25
Wavelength of the guidance laser, μm	0.65
Diameter of the light aperture in the optical connector, mm	0.22
Time mode of operation	continuous, pulsed or pulse-periodic
Pulse duration, ms	from 1 to 1000
Pause duration, ms	from 1 to 1000
Beam divergence at the fiber output	25°
Optical connector type	SMA
Length of the fiber tool fiber, m, not less than	2
Light transmittance of the fiber tool, %, not less than	60
Supply voltage, V	220 ± 10%
Network frequency, Hz	50
Power consumption, VA, not more than	200
Dimensions, mm, not more than	360 × 250 × 160
Weight, kg, not more than	7

**Table 2:** Distribution of oral mucosal samples of rabbits by study groups.

Time Point of the Experiment	Study Group			Rabbits (*n*)
	Mode #1	Mode #2	Mode #3	
1 day/samples	5	5	5	8
14 day/samples	5	5	5	8
28 day/samples	5	5	5	8
42 day/samples	5	5	5	8
Total	20	20	20	32

**Table 3:** Scoring system for evaluating histological features of exudation in the laser treatment area.

Score	Histological Features
0	No exudation
1	Slight edema (small amount of fluid in the intercellular space; pale blue staining of collagen fibers with Mallory)
2	Mild edema (moderate amount of fluid in the intercellular space; minor foci of matrix homogenization)
3	Significant edema (large amount of fluid in the intercellular space; matrix homogenization, collagen fiber fibrillation)

**Table 4:** Scoring system for evaluating histological features of inflammatory infiltration in the laser treatment area.

Score	Histological Features
0	No inflammation
1	Infiltration with singular immune cells (less than 10 cells in the field of view at a magnification of ×400)
2	Moderate level of infiltration with immune cells (from 11 to 29 cells in the field of view at a magnification of ×400)
3	High level of infiltration with immune cells (more than 30 in the field of view at a magnification of ×400)

**Table 5:** Scoring system for evaluating the histological features of neoangiogenesis in the laser treatment area.

Score	Histological Features
0	No signs of newly formed blood vessels
1	The beginning of vessel formation: the vascular wall is absent; the endothelium is represented by a thin layer of endotheliocytes
2	Continued vessel formation: t. adventitia is absent, muscle fibers in t. media are thin, the endothelium is of normal structure
3	The blood vessels are formed: the wall has a three-layer structure (t. adventitia, media, intima), the endothelium is of normal structure

**Table 6:** Scoring system for assessing the expression of antibodies against α-SMA.

Score	Histological Features
−	No expression of α-SMA
+	Singular positive cells
++	Moderate level of positively stained cells (less than 19 per field of view at 400× magnification)
+++	High level of positively stained cells (more than 20 per field of view at 400× magnification)

## Data Availability

The original contributions presented in this study are included in the article. Further inquiries can be directed to the corresponding author.

## Abbreviations

The following abbreviations are used in this manuscript:

α-SMA	α-smooth muscle actin
H&E	hematoxylin and eosin
